# Locking plate for treating traumatic sternoclavicular joint dislocation: a case series

**DOI:** 10.1186/s12891-017-1903-8

**Published:** 2018-01-09

**Authors:** Rongguang Ao, Yalong Zhu, Jianhua Zhou, Zhen Jian, Jifei Shi, Cheng Li, Wankun Hu, Baoqing Yu

**Affiliations:** Department of Orthopedics, Shanghai Pudong Hospital, Fudan University Pudong Medical Center, 2800 Gongwei Road, Huinan Town, Pudong, Shanghai, 201399 People’s Republic of China

**Keywords:** Sternoclavicular joint, Dislocation, Open reduction and internal fixation

## Abstract

**Background:**

Traumatic sternoclavicular joint dislocations are rare; closed reduction is the primary treatment. The failure of closed reduction or a prominent insult to the skin may require surgery to ensure the best possible outcome.

**Methods:**

The records of 5 patients operated at our institution for sternoclavicular joint dislocation were reviewed. All patients were treated with open reduction and single 3.5-mm locking plate was used for fixation. Outcomes were evaluated with the Constant Shoulder Score (CSS) and Disability of the Arm, Shoulder, and Hand (DASH) questionnaire. Intraoperative and postoperative complications were recorded.

**Results:**

All the patients had an average follow-up of 14 months (range, 11–16 months). At the final follow-up, the mean CSS score was 89.5 (range, 78–98) and the mean DASH score was 9.0 (range, 4–16). There were no early complications, including wound infection or neurologic or vascular deficits; there were also no broken or loosened screws or plates. No case of redislocation or arthrosis was observed.

**Conclusion:**

Our study indicates that open reduction and fixation with a single locking plate for the treatment of traumatic sternoclavicular joint dislocation is a safe, relatively simple surgical procedure that can lead to satisfactory outcomes.

## Background

Sternoclavicular joint dislocations (SCJ) are rare, accounting for approximately 3% of injuries to the shoulder girdle [1]. Some 90% to of 95% SCJ dislocations are anterior [2], and most can be treated with closed reduction. Once recurrence or instability of the anterior SCJ dislocation is noted, the prominence of the medial clavicle may cause discomfort, and operative management may become necessary [3]. Posterior SCJ dislocations are life-threatening injuries because of their potential for causing mediastinal compression, compression of the brachial plexus, pneumothorax, respiratory distress, as well as vascular injuries [4]. Prompt closed reduction is recommended for posterior dislocations of the SCJ. If closed reduction fails, operative management is recommended [3].

Many surgical techniques have been used to treat unstable or chronic SCL dislocations, including osteosynthesis with pins [5] and K-wires [6], plate fixation [7–9], and ligament reconstruction [10–13]. But only three papers have reported on plate fixation for the treatment of SCJ dislocations, using three different types of implants: Balser plates [7], standard 3.5-mm LC/DCP with a ledge-plating technique [8], and a dual locking plate [9]. Good results were achieved with these techniques, but plate fixation has not been widely used.

In this paper, we report on the operative technique and outcomes of using a single locking plate to treat traumatic SCJ dislocation.

## Methods

### General data

Institutional review board approval for the study was obtained. A retrospective study that included all 8 cases of SCJ dislocation treated between October 2008 and December 2015 was performed. The primary management is closed reduction according to recommended reduction methods [3]. The indications for proceeding to surgery were as follows: (1) dislocations that could not be reduced by conservative management and (2) dislocations that appeared vulnerable to recurrence with movement of the shoulder joint, and cases in which there was a prominent insult to the skin. Five patients who met the criteria for surgery were treated with open reduction and internal fixation using a single 3.5-mm locking plate; these cases included three anterior and two posterior dislocations. The age, gender distribution, affected side, cause of the injuries, dislocation type, associated injuries, and duration of follow-up are listed in Table 1.

**Table 1:** General conditions

Identifier	Gender/Age (y)	Affected side	Cause	Dislocation type	Associated injury	Follow-up (months)	DASH score/Constant score
Patient 1	F/29	L	Traffic accident	Anterior	No	16	4/98
Patient 2	M/31	R	Sport injury	Posterior	No	11	6/96
Patient 3	F/43	R	Traffic accident	Anterior	Ipsilateral acromioclavicular joint dislocation (Rockwood type IV), left proximal humeral fracture	15	16/78
Patient 4	M/37	L	Traffic accident	Posterior	No	15	10/87.5
Patient 5	F/41	R	Traffic accident	Anterior	No	13	9/88

### Surgical technique and rehabilitation

Surgery took place with the patient under general anesthesia and in the supine position. A transverse incision was made over the medial clavicle and sternum. The skin and subcutaneous tissues were dissected and, if possible, the platysma was incised and elevated as a separate layer. The periosteum of the medial clavicle was reflected superiorly and inferiorly, at which point the injured ligaments of the SCJ could be identified.

**Fig. 1:**
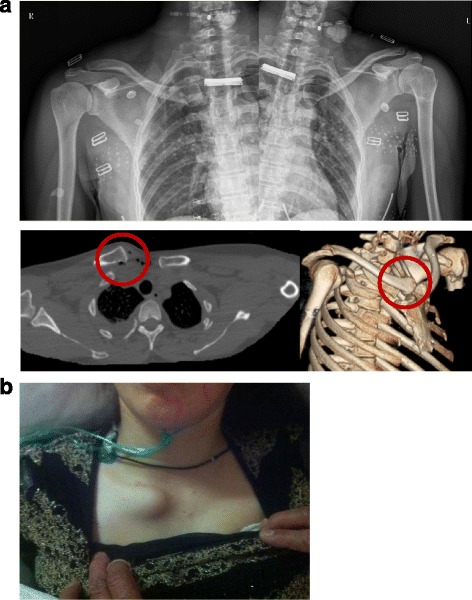
In patient 3, a 43-year-old female, the preoperative x-ray and computed tomography scan (**a**) show a right anterior dislocation of the sternoclavicular joint and a left proximal humeral fracture. A preoperative photograph (**b**) showing an obvious prominence at the medial end of the clavicle

**Fig. 2:**
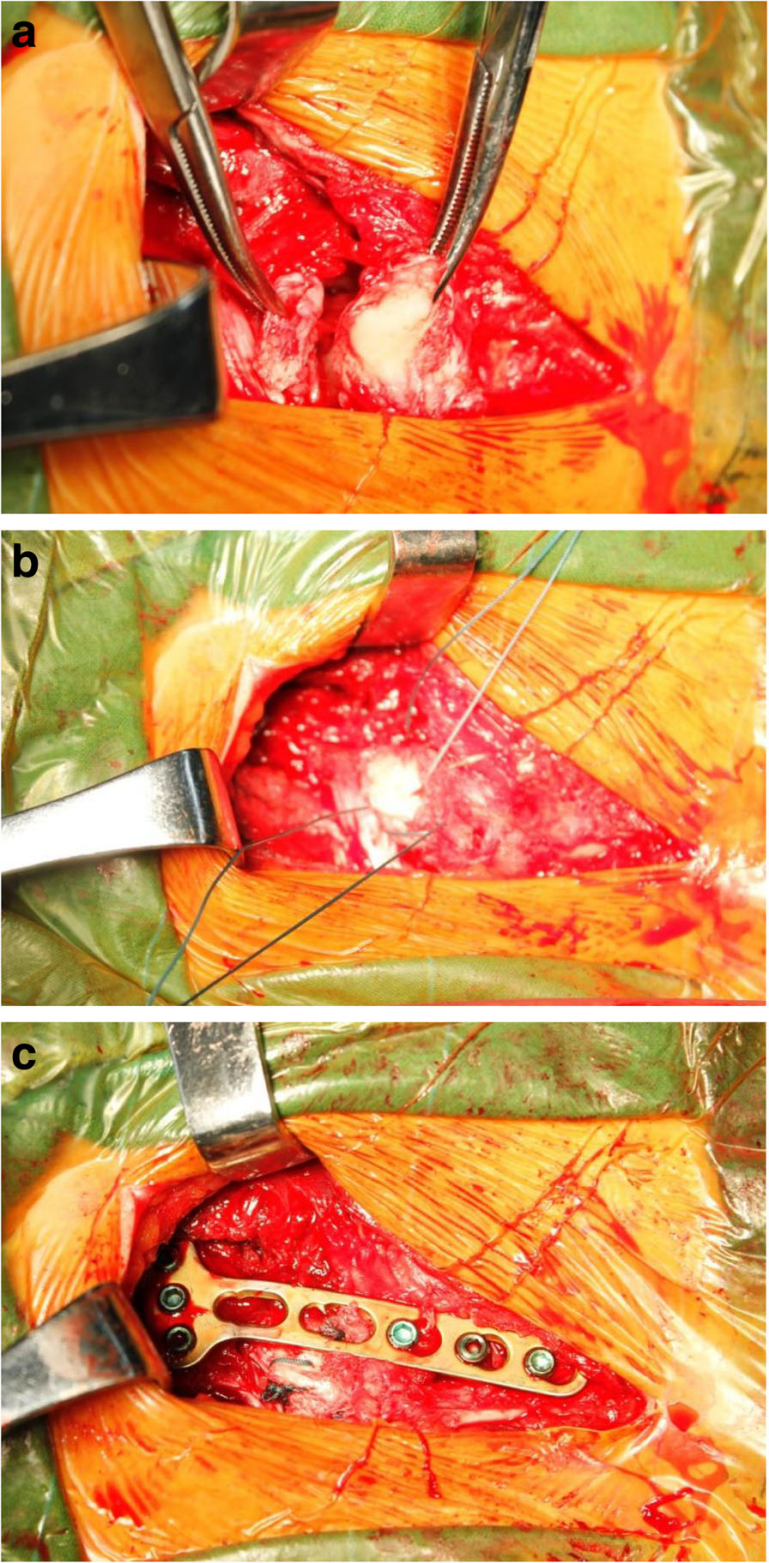
Intraoperative **a** the anterior ligament was torn. **b** nonabsorable suture was used to repair the torn ligament and **c** the 3.5-mm locking plate placed anteriorly with three screws in the manubrium and three in the clavicle

In the case of the anterior dislocations (Fig. 1), we found that the anterior capsule and ligaments were torn (Fig. 2a). After manual reduction of the SCJ, the medial end of clavicle would move anteriorly, indicating instability. Therefore we maintained reduction of the SCJ and used nonabsorbable suture to repair the torn ligaments and capsule (Fig. 2b), finally applying the locking plate for fixation. The plate was placed on the anterior part of the sternum and medial clavicle. First fixation at the medial clavicle was performed, using a short drill to protect the vascular structures (subclavian artery and vein). Bicrotic locking screws were used to maintain reduction of the SCJ. Then the anterior cortex of the sternum was carefully drilled and unicortical locking screws placed (Fig. 2c).

**Fig. 3:**
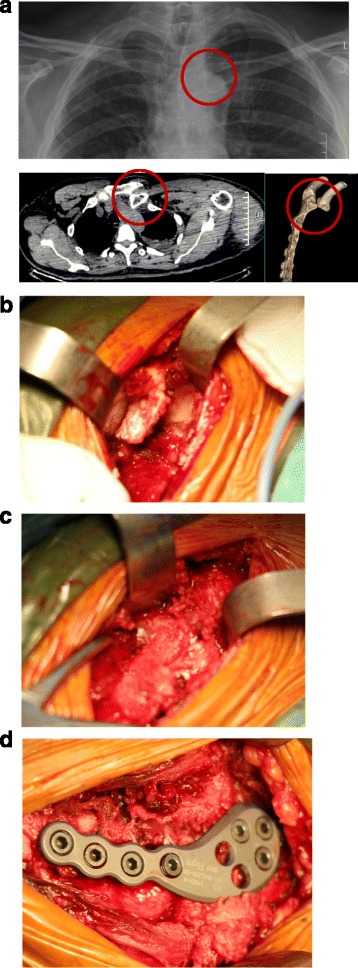
In patient 2, a 31-year-old male, the preoperative x-ray and computed tomography scan **a** show a posterior dislocation of the sternoclavicular joint. Intraoperative **b** showing the exposed articular surface of the clavicle from the sternum side; and the position of the medial clavicle was not visible, **c** showing the medial end of the clavicle being reduced with forceps, and **d** showing the 3.5-mm locking plate placed anteriorly with three screws in the manubrium and four in the clavicle

**Fig. 4:**
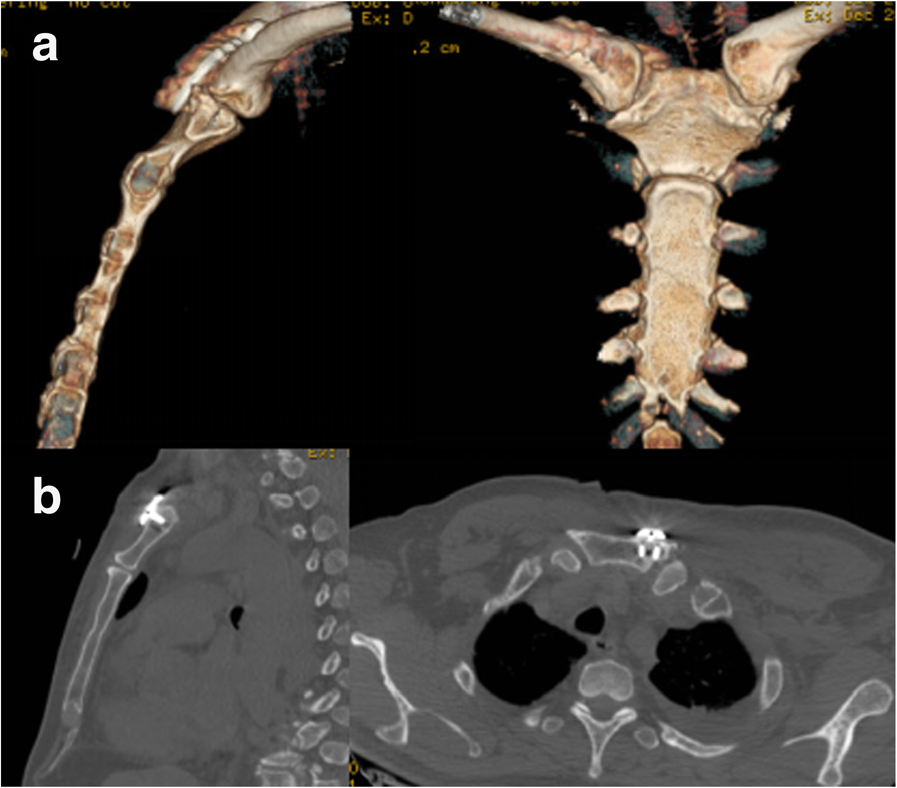
Postoperative A postoperative three-dimensional computed tomography scan **a** showing good reduction and fixation with the locking plate, sagittal and transverse computed tomography **b** scans showing unicortical screw fixation in the sternum

For posterior dislocations (Fig. 3a), we dissected the anterior ligament and capsule to expose the articular surface of the clavicle from the sternum side. Because the position of the medial clavicle was not visible (Fig. 3b), we identified the clavicle from the lateral side and then moved to the medial end of the clavicle. The injured upper limb was retracted with shoulder abduction to about 90 degrees. Reduction was then carefully held with forceps (Fig. 3c). A locking plate was used for fixation in the same manner as with the anterior dislocation (Figs. 3d and 4). Nonabsorbable suture was used to repair the ligament and capsule before plating.

After surgery, the injured extremity was immobilized in a sling for 3 weeks, during which active range-of-motion exercises for the elbow and shoulder were encouraged. Three weeks after operation, passive mobilization of the shoulder was increased while gradually transitioning to active exercises.

All patients were asked to follow up monthly for 3 months after their operations, and then every 3 to 6 months after implant removal. Functional evaluation was implemented using the Constant Shoulder Score (CSS) and Disability of the Arm, Shoulder, and Hand (DASH) questionnaire. Documented postsurgical complications included infection, implant failure, and recurrent dislocation. All plates were removed after 6 months to avoid breakage or loosening of the plate and screws.

## Results

The average follow-up was 14 months (range, 11–16 months). The mean age, gender distribution, dislocation type, and associated injuries are shown in Table 1. All patients had secondary operations for plate removal 6 months postoperatively. At the final follow-up, the mean CSS and DASH scores were 89.5 (range, 78–98) and 9.0 (range, 4–16), respectively (Table 1).

There were no early complications, including wound infection or neurologic or vascular deficit; also, no screws or plates were broken or loosened. No case of redislocation or arthrosis was observed. Four patients were satisfied with the outcomes. All patients were able to return to their previous activities.

**Fig. 5:**
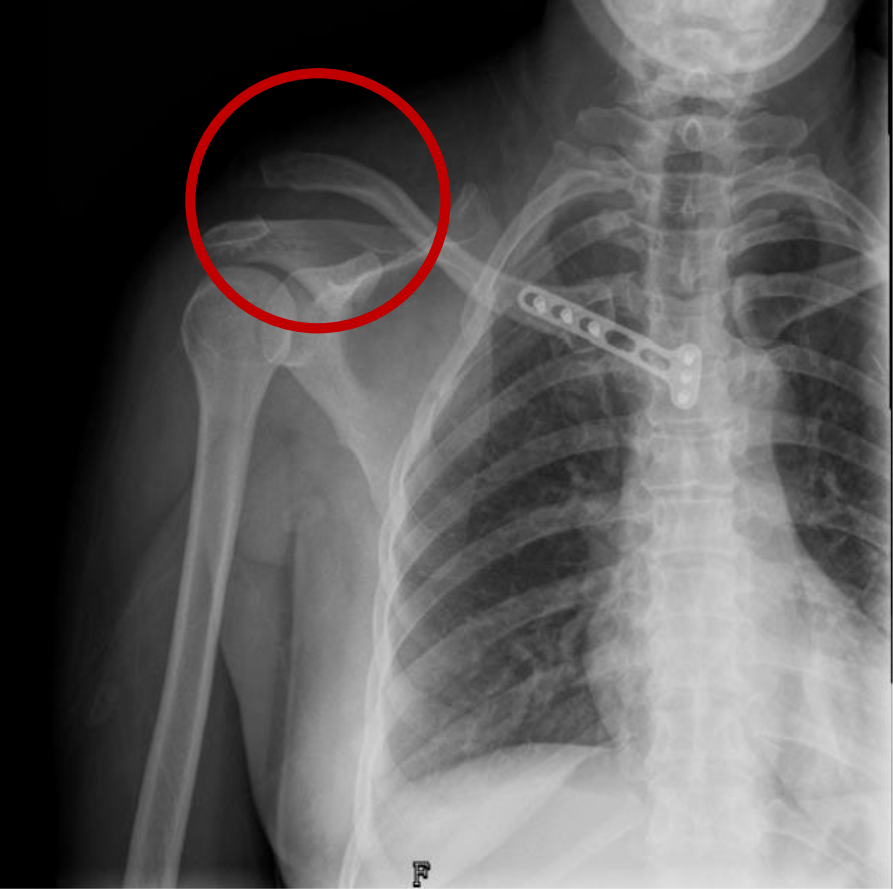
A postoperative x-ray showing right acromioclavicular joint dislocation

**Fig. 6:**
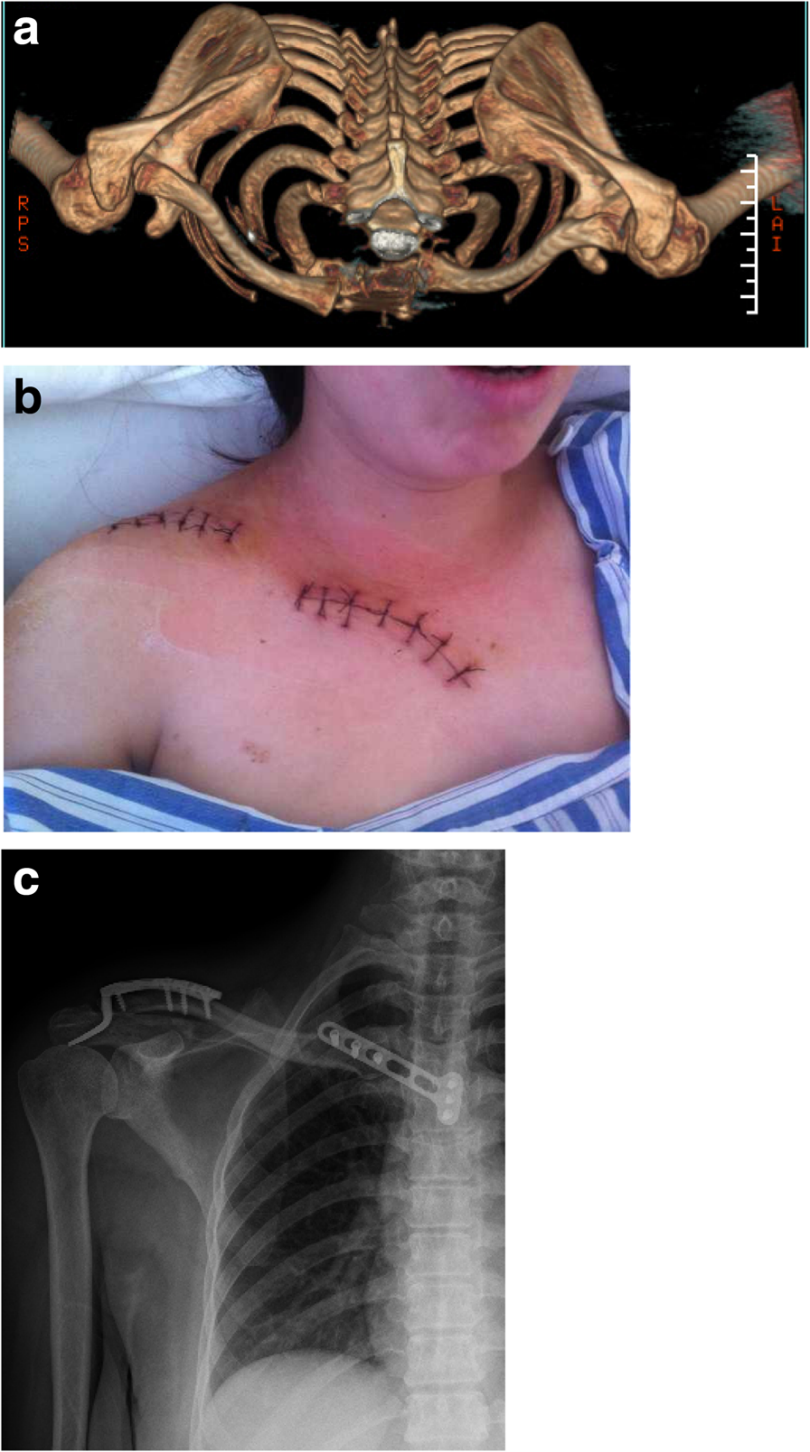
Preoperative **a** Initial transverse and sagittal computed tomography scans of both sides of the shoulder girdle showing a posterior acromioclavicular joint dislocation (Rockwood type IV) and an anterior sternoclavicular joint dislocation, **b** The final operative incision and **c** postoperative x-ray

Only patient 3 had an unsatisfactory outcome. She had a right floating clavicular injury and a left proximal humeral fracture. She was initially diagnosed with a right anterior SCJ dislocation and left proximal humeral fracture (Fig. 1). Closed reduction for the anterior SCJ dislocation failed and open reduction and internal fixation for the two injuries were performed on the following day. The postoperative x- ray showed that the right acromioclavicular joint (ACJ) was dislocated—that is, the Rockwood type V (Fig. 5). After reviewing the initial CT scan again, we found that posterior ACJ dislocation was actually a Rockwood type IV (Fig. 6a). We therefore performed a second operation to treat the floating clavicle (Fig. 6b). The screws previously placed in the clavicle were removed and simultaneously reduction of the ACJ and SCJ dislocations was implemented (Fig. 6c). A hook plate was used to treat the ACJ dislocation. Three months after the second operation, the two implants for treating the floating clavicle were removed. At the last follow-up, although there was no pain or redislocation, abduction of the injured shoulder was limited to 110 degrees.

## Disscussion

The SCJ is a saddle-type joint that represents less than half of the medial clavicle as it articulates with the upper angle of the sternum [1]. When the shoulder girdle moves, the SCJ has some range motion in three planes [1]. Despite its intrinsic instability owing to its bony anatomy, strong soft tissues stabilize the structures—including the ligaments, subclavius muscle, articular disc, and capsule—such that the SCJ is one of most rarely dislocated joints in the body [1].

Indirect force from shoulder girdle is the common mechanism of SCJ dislocation [3]. Occasionally, a direct force applied to the medial clavicle will lead to a posterior dislocation [3]. When the prominence of the medial clavicle is inspected after trauma, the anterior SCJ dislocation should be taken into account; a palpable defect of the medial end of the clavicle is the obvious sign of a posterior dislocation of the SJC [3]. After such a diagnosis, it is important that the entire clavicle and AC joint be examined. Especially for the anterior SCJ dislocation, the ACJ should be checked carefully to determine whether or not there is a posterior dislocation [14]. In the case of patient 3, we neglected to examine the ipsilateral ACJ and diagnosis of the floating clavicle was delayed.

Because it may be blocked by artifacts or neighboring structures, dislocation of the SJC can easily be missed on x-ray. A CT scan is the best diagnostic imaging method and can serve to distinguish between medial clavicular fractures, physeal separations, and SCJ dislocations. Careful scrutiny of a three-dimensional CT scan can serve to determine whether there is displacement of the SCJ and whether or not there is also a posterior AC dislocation.

Closed reduction is the primary choice for the treatment of an SCJ dislocation [15, 16]. For traumatic anterior SCJ dislocations, although there is a risk of recurrent instability, functional deficits after closed reduction have rarely occurred [3]. However, if the medial end of the clavicle clavicle is prominent, surgical correction may be preferred [3]. For traumatic posterior SCJ dislocations, there may be more potential complications; therefore such injuries should immediately be treated with closed reduction. When the posterior SCJ dislocation cannot be reduced in this way, an open reduction should be performed urgently to minimize the risk of cardiovascular compromise [3].

The objective of surgery is to restore the bony anatomy of the SCJ and restore stability to the joint. The risk in the intraoperative process is that of damaging the neighboring structures in the course of drilling holes in the sternum and the medial clavicle and also that of causing loose or unstable implants to migrate postoperatively. The optimal surgical process should minimize these risks as far as possible.

There are many operative methods to treat SCJ dislocation, including ligament repair with reconstruction [10–13], Kirschner wire or pin fixation [5, 6], as well as plate fixation [7–9]. The best choice among the various procedures is also controversial. Ligament repair with reconstruction is the most common method according to the literature [10–13]. These procedures require relatively complex operative manipulation, greater soft tissue dissection, and an extended time of postoperative immobilization. Kirschner wire or pin fixation is contraindicated owing to the associated high risk with their migration into vital structures [5, 6].

Up to now, three studies of plate fixation for the treatment of SCJ dislocation have been reported, including that of Franck et al., who utilized Balser plates for treating three posterior SCJ dislocation [7]. Shuler and Pappas used dual perpendicular locking plates to fix two posterior dialocations [8], and Hecox et al. used a ledge plating technique to treat two posterior dislocation [9]. The Balser plate requires a hook insert into the sternum, which appears to put vital structures at risk. Dual locking plates can achieve rigid fixation for the SCJ, but owing to additional soft tissue manipulation, medical cost will be greater. The ledge plating technique, which does not require the use of a drill or screws into the sternum, can obviously avoid damage to the vital structures, but the stability of fixation of the SCJ may be insufficient. The biomechanics involved in this innovative method requires further research.

Use of a single locking plate may be a preferable alternative for treating SCJ dislocations. The purpose of the locking plate is to maintain the SCJ reduction and allow the soft tissues around the joint to heal. In the surgical process, unicortical screws in the sternum and biocortical screws in the medial clavicle were used to maintain the stability of the SCJ. Meanwhile, we sutured the injured ligaments and capsules to provide preliminary stability to the joint. After stable fixation of the locking plate, the soft tissues can heal and the SCJ will be stable. In order to avoid iatrogenic injury, only the anterior cortex of the sternum was drilled and bicortical screws were used for fixation. Finally, the locking plate allows relatively stable fixation as well as a certain degree of movement around the fracture fragment. Thus, in utilizing a single locking plate to treat the SCJ dislocation, we can also ensure a certain degree of joint movement, which facilitates healing and the recovery of shoulder function.

We believe that single locking plate fixation combined with repair of the ligament and capsul is easier to manipulate, minimizes the manipulation of soft tissues, and protects the periosteal blood supply. Unicortical drill and screw fixation in the sternum is a relatively safe operative process in terms of protecting the vital structures. Most of these patients have good function without recurrent dislocation or subluxation.

To avoid the migration of loose or broken implants, the locking plates must be removed about 3 months after surgery; that is the disadvantage of this technique.

Furthermore, this study has some limitations. It is a retrospective study involving a small number of patients with a short-term follow-up. A long-term follow-up would be needed to determine whether postoperative arthritis developed in these patients.

## Conclusion

Fixation using a single locking plate combined with repair of the ligament and capsule is relatively easy to do; it decreases the risk of the soft tissues injury and protects the periosteal blood supply. Our study indicates that open reduction and single-locking-plate fixation for the treatment of traumatic sternoclavicular joint dislocations is a safe, relatively straightforward surgical procedure that can lead to satisfactory outcomes.

## Abbreviations

ACJ: Acromioclavicular joint
CSS: Constant Shoulder Score
DASH: Disability of the Arm, Shoulder, and Hand
SCJ: Sternoclavicular joint
